# Supplementary material for: ORAI2 Down-Regulation Potentiates SOCE and Decreases Aβ42 Accumulation in Human Neuroglioma Cells
Source: Int J Mol Sci. 2020 Jul 25;21(15):5288. doi: 10.3390/ijms21155288 (PMC7432374; doi:10.3390/ijms21155288)
Supplement: Supplementary file 1 [file ijms-21-05288-s001.pdf]

Supplementary Materials

Preparation of protein extracts and Western blot analysis

H4 cells overexpressing ORAI2-myc and ORAI2-myc-mVenus were lysed in RIPA buffer (50 mM Tris, 150 mM NaCl, 1% Triton X-100, 0.5% deoxycholic acid, 0.1% SDS, protease inhibitor cocktail, pH 7.5) and incubated on ice for 30 min. The lysate was cleared by centrifugation material was spun down at 4000 *g* for 5 min at 4°C. 50 µg of protein were loaded onto precast polyacrylamide gels (10%) and immunoblotted with anti-ORAI2 (Alomone ACC-061), anti-myc (Millipore 05724) and anti-actin (Sigma-Aldrich A4700). The proteins were visualized by chemiluminescence using as a secondary antibody anti-mouse (Bio-Rad, 1:5000) or anti-rabbit (Bio-Rad, 1:3000). Densitometry was performed using ImageJ software (National Institutes of Health).

Quantitative real-time PCR

RNA was extracted from H4-APPswe cells transfected with ORAI2-, ORAI1- or control-siRNAs using the NucleoSpin RNA purification kit (Macherey-Nagel), followed by cDNA synthesis with SuperScript II Reverse Transcriptase (Invitrogen, 18064071). PCR amplification was performed in an iQ5 Thermal Cycler (BioRad), using SYBR Green Supermix (BioRad) as reagent. For each cDNA, the efficiency of the reaction was estimated with a standard curve by using the cDNA reverse transcript from non-transfected H4-APPswe cells. The transcript of GAPDH, cypA and B2M were used as internal controls. The following primers (Invitrogen) were employed:

cypA: 5’-TTCATCTGCACTGCCAAGA-3’ and 5’-TCGAGTTGTCCACAGTCAGC-3’;

B2M: 5’-TATCCAGCGTACTCCAAAGA-3’ and 5’-GACAAGTCTGAATGCTCCAC-3’;

GAPDH: 5’-CACCATCTTCCAGGAGCGAG-3’ and 5’-TTCACACCCATGACGAACAT-3’;

ORAI1: 5’-AGTTACTCCGAGGTGATGAGC-3’ and 5’-TAGTCGTGGTCAGCGTCCAG-3’;

ORAI2 primer-A:

5’-TGAGCAACATCCACAACCTGA-3’and 5’-GGGAGGAACTTGATCCAGCAG-3’;

ORAI2 primer-B:

5’- GATGGAGAGCCTGAGTTGGC-3’ and 5’ GGTCGATAGGCACGTTAAGC-3’.

Data are expressed as percentage ratio change relative to the internal control transcript.

Immunocytochemistry

H4 cells expressing ORAI2-myc, ORAI2-myc-mVenus, ER- and rab5-mCherry or HEK293T cells expressing ER-GFP were fixed in Phosphate Buffered Solution (PBS) containing 4% paraformaldehyde for 15 minutes, incubated with NH_4Cl (50 mM) for 20 min, permeabilized with 0.1% Triton X-100 in PBS for 3 minutes and then blocked with 2% BSA and 0.2% gelatine for 30 minutes. ORAI2 was stained using anti-ORAI2 (Sigma-Aldrich PRS4111) and myc-tag with anti-myc (Millipore 05724). For localization studies, Rab5, early endosome antigen 1 (EEA1) and lysobisphosphatidic acid (LBPA) were stained with anti-rab5

(Synaptic Systems 108011), anti-EEA1 (BD Bioscience 610456) and anti-LBPA (kindly provided by J. Gruenberg, University of Geneva, Switzerland). Alexa Fluor 555 and 488 secondary antibodies (Thermo Fisher Scientific) were applied for 1 hour at room temperature. Coverslips were mounted using Mowiol (Sigma-Aldrich).

Confocal analysis

HEK293T and H4 cells expressing ORAI2 or stained with the specific antibody, were analysed with a Leica TCS-SP5-II confocal system equipped with a Plan Apo 100X objective (NA 1.4). For all images, the pinhole was set to 1 Airy unit. Acquisition was performed at 1024×1024 pixels per image at 200 Hz. Co-localization analysis was performed on z-stacks acquired with 0.5 μm steps. The green and red channel images were acquired independently, and the photomultiplier gain for each channel was adjusted to minimize background noise and saturated pixels and maintained among different sets of experiments. Analyses were carried out on single-plane images using ImageJ plug-ins. A background region of interest (ROI) was selected, identical for the two channels, and the corresponding backgrounds were subtracted through an ImageJ ROI plug-in. Subsequently, ROIs were drawn corresponding to the cell profiles, as defined by the external perimeter of the investigated marker and applied to the ORAI2 channel. The Pearson's coefficient was calculated applying the ImageJ co-localization analysis plug-in.

Aβ ELISA

The day before the assay, the cell culture medium was changed with 1 ml of fresh Neurobasal medium (ThermoFisher 21103049) containing 2% B-27 supplement (ThermoFisher 17504044). The 24-h-conditioned media were then mixed with 0.1 ml of 10X solution of phosphatase and protease inhibitors (cOmplete, Mini, protease and PhosSTOP phosphatase inhibitor cocktail, Roche) in RIPA buffer and frozen at -80ºC. Aβ40 and Aβ42 levels were quantified with Sensolyte® Anti-Human Aβ40 and Aβ42 ELISA from ANASPEC (#AS-55551 and #AS-55552, respectively).

SUPPLEMENTARY FIGURES

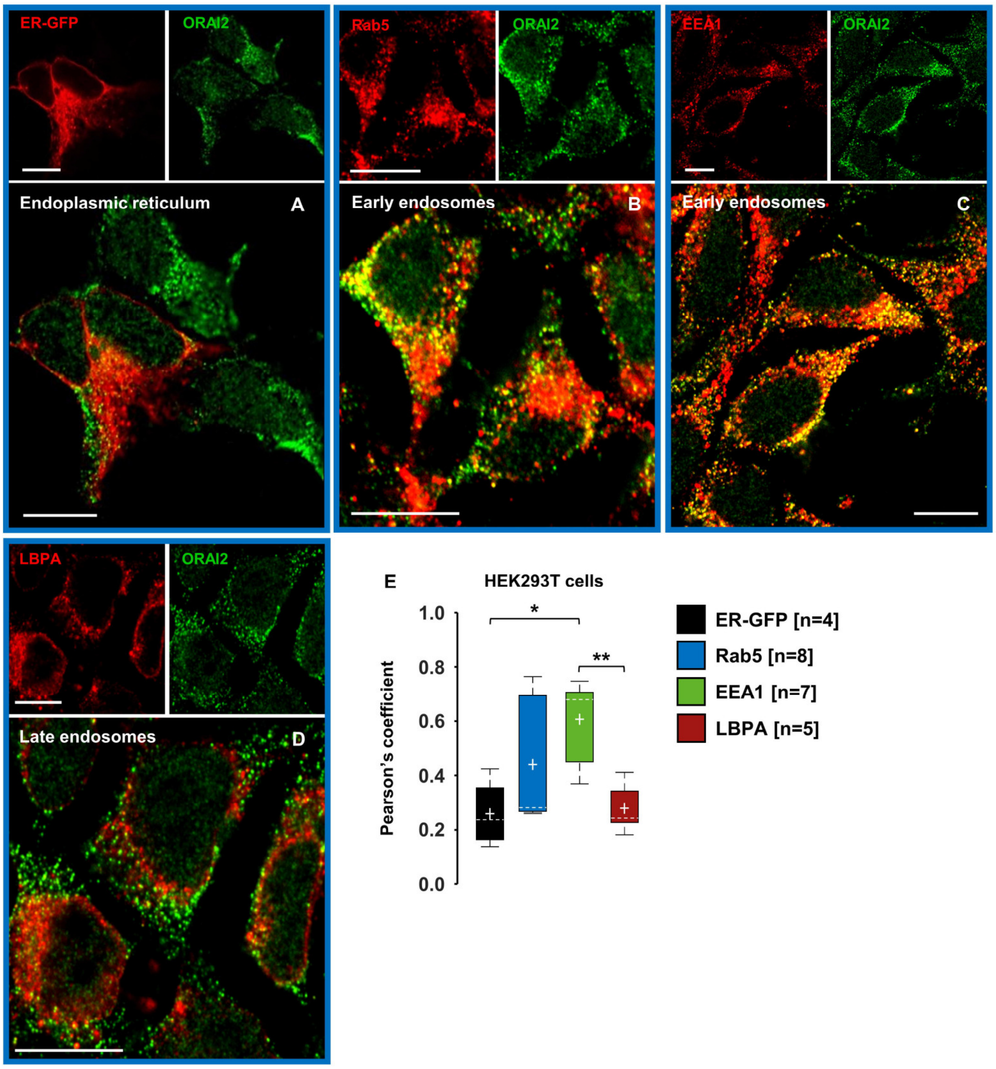

Figure S1. Endogenous ORAI2 localizes to the early endosomal compartment in HEK293T cells. Confocal microscope immunofluorescence analysis of the subcellular localization of endogenous ORAI2 in HEK293T cells. Cells, were stained for endogenous ORAI2 (green, right panels) and either transfected or stained with different subcellular markers (red, left panels); image overlay (bottom panels): cells expressing ER-GFP (**A**) or stained for the early endosome markers rab5 (**B**) and EEA1 (**C**), and for the late endosome marker LBPA (**D**). Representative images of three independent experiments. Signal co-localization was estimated using Pearson's coefficient (**E**): 0.26 ± 0.07 (ER); 0.44 ± 0.09 (rab5); 0.61 ± 0.07 (EEA1) and 0.28 ± 0.04 (LBPA) (n = number of coverslips from three independent experiments, Wilcoxon-Mann-Whitney test, * $p < 0.05$; ** $p < 0.01$), bars, 10 μm.

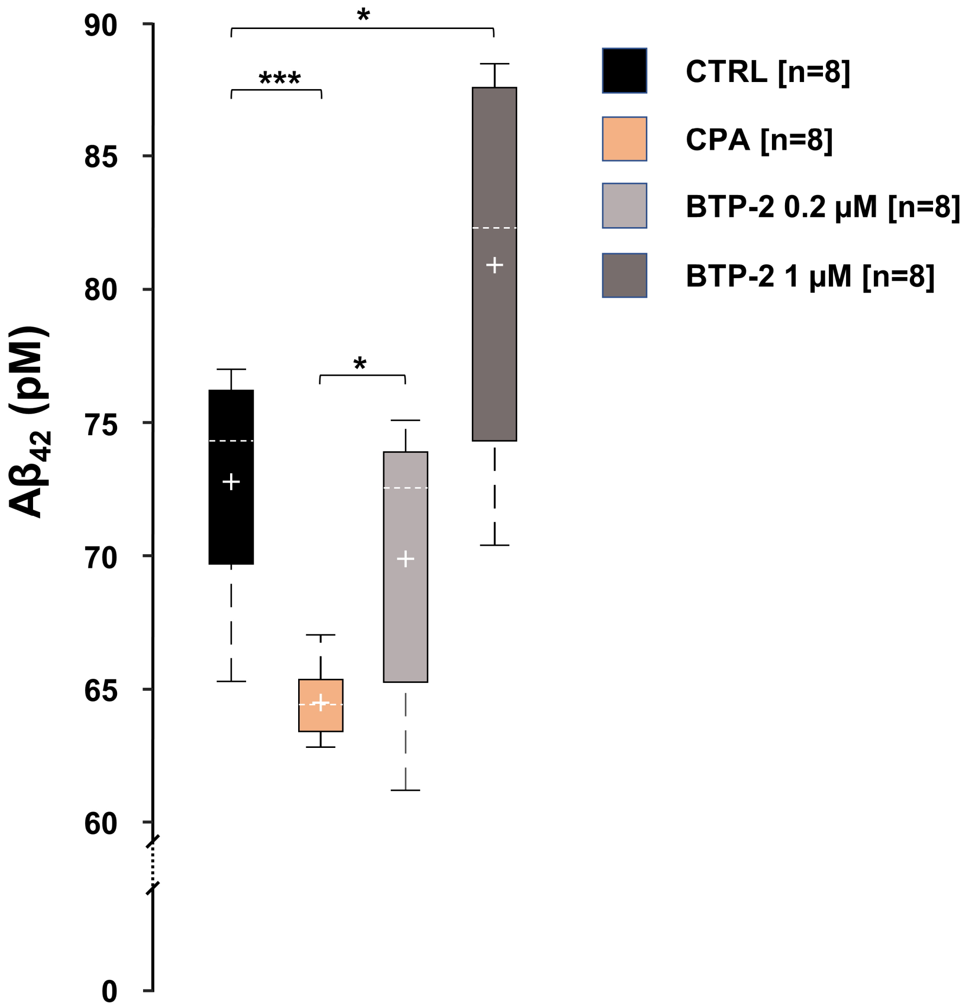

Figure S2. Inverse relationship between SOCE modulation and Aβ42 secretion. H4-APPswe cells were treated for 24 hours with: DMSO (0.1%) (CTRL), CPA (0.5 µM), BTP2 (0.2 µM or 1 µM) in complete culture medium. Conditioned media were collected with protease and phosphatase inhibitors and quantified with an Aβ42 ELISA kit (see Supplementary Materials). The Aβ42 secretion was decreased by chronic SOCE activation with CPA (0.5 µM) and increased by SOCE inhibition with BTP2 (1 µM) (n = number of samples, Wilcoxon-Mann-Whitney test, ** $p < 0.01$, * $p < 0.05$).

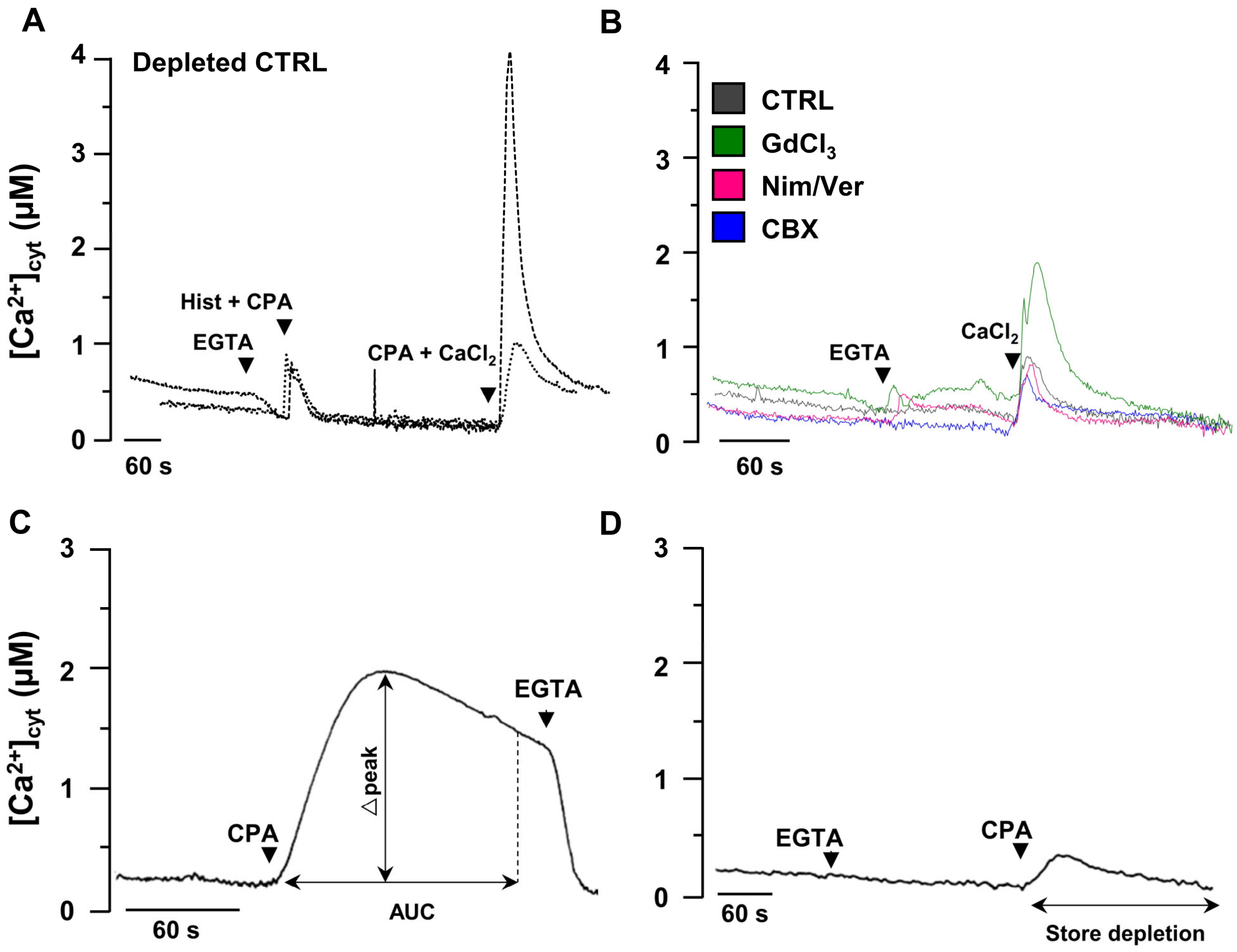

Figure S3. SOCE protocols in H4-APPswe cells. (A) Very heterogenous Ca^{2+} rises occurred in H4-APPswe cells upon store depletion induced by histamine (Hist, 100 µM) and CPA (20 µM) in a Ca^{2+}-free mKRB containing EGTA (0.6 mM). **(B)** Large Ca^{2+} rises also occurred upon $CaCl_2$ (1 mM) addition to cells only exposed to Ca^{2+}-free mKRB containing EGTA (0.6 mM) (black). These Ca^{2+} rises were not abolished by $GdCl_3$ (1 µM, green), nimodipine (Nim, 1 µM) plus verapamil (Ver, 20 µM, pink) and carbenexolone (CBX, 50 µM, blue), known inhibitors of SOCE, voltage-operated Ca^{2+} channels and gap-junctions, respectively. (**C**) In H4-APPswe cells SOCE was induced by CPA addition in mKRB containing $CaCl_2$ (1 mM); SOCE level was estimated by the peak over basal (Δpeak) and the area under the curve (AUC). (**D**) The store contribution to Ca^{2+} rise was evaluated in parallel experiments by CPA addition in a Ca^{2+}-free, EGTA-containing medium.

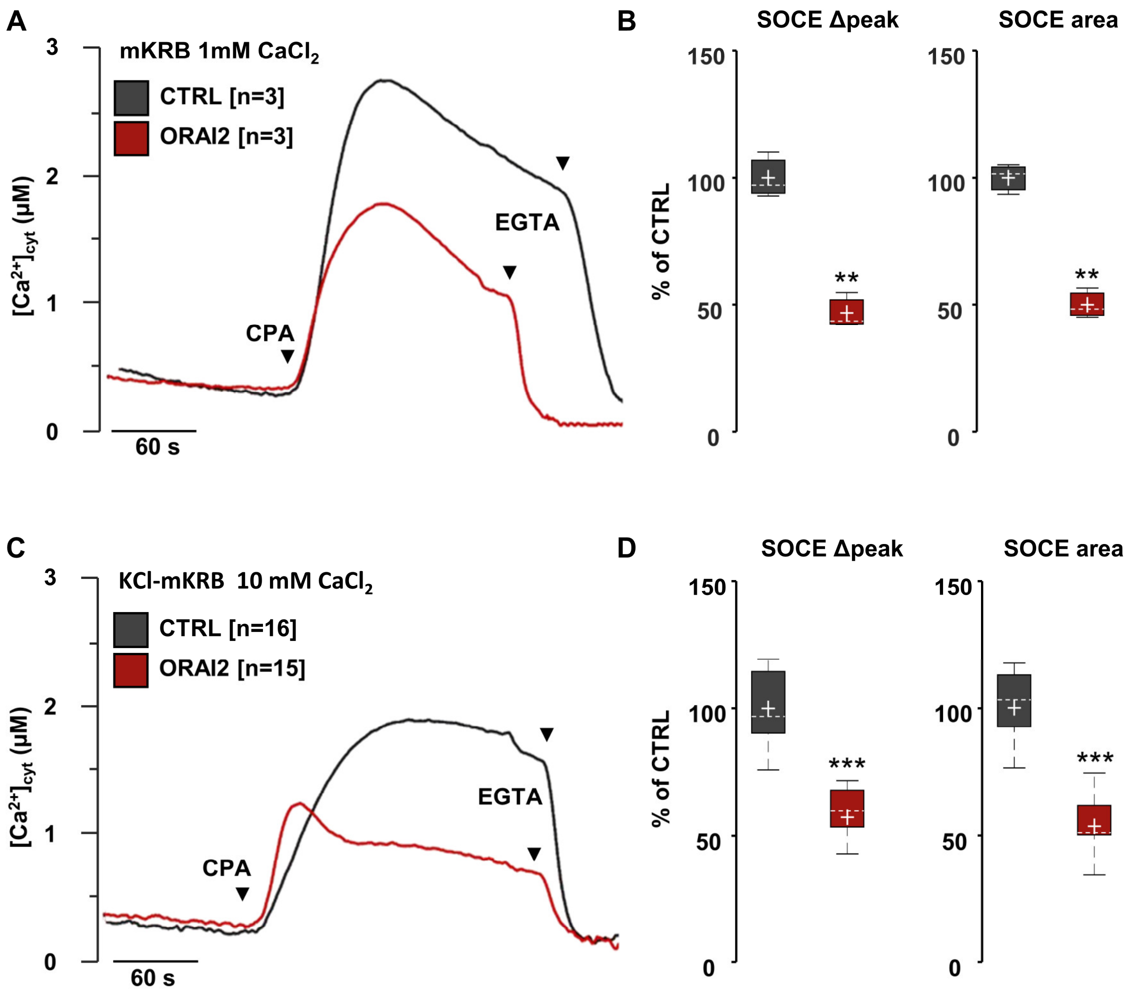

Figure S4. ORAI2 overexpression reduces SOCE in H4-APPswe. Cells were co-transfected with the cDNA coding for ORAI2-myc (or with the void-vector, CTRL), plus STIM1 and cytAEQ in a 2:6:1 ratio. After 24 hours, cells were bathed at 37°C, for two minutes in mKRB (**A,B**) or KCl-mKRB (**C,D**) containing 1 or 10 mM $CaCl_2$, respectively. Cells were then treated with CPA (20 µM) to activate SOCE. (**A,C**) Representative traces for CTRL (black) and ORAI2-overexpressing cells (red) respectively. (**B,D**) Boxplots of SOCE Δpeak and AUC, measured within 2 minutes, upon baseline subtraction. Data are expressed as percentage of control (CTRL), n = number of coverslips, Wilcoxon-Mann-Whitney test, ** $p < 0.01$; *** $p < 0.001$.

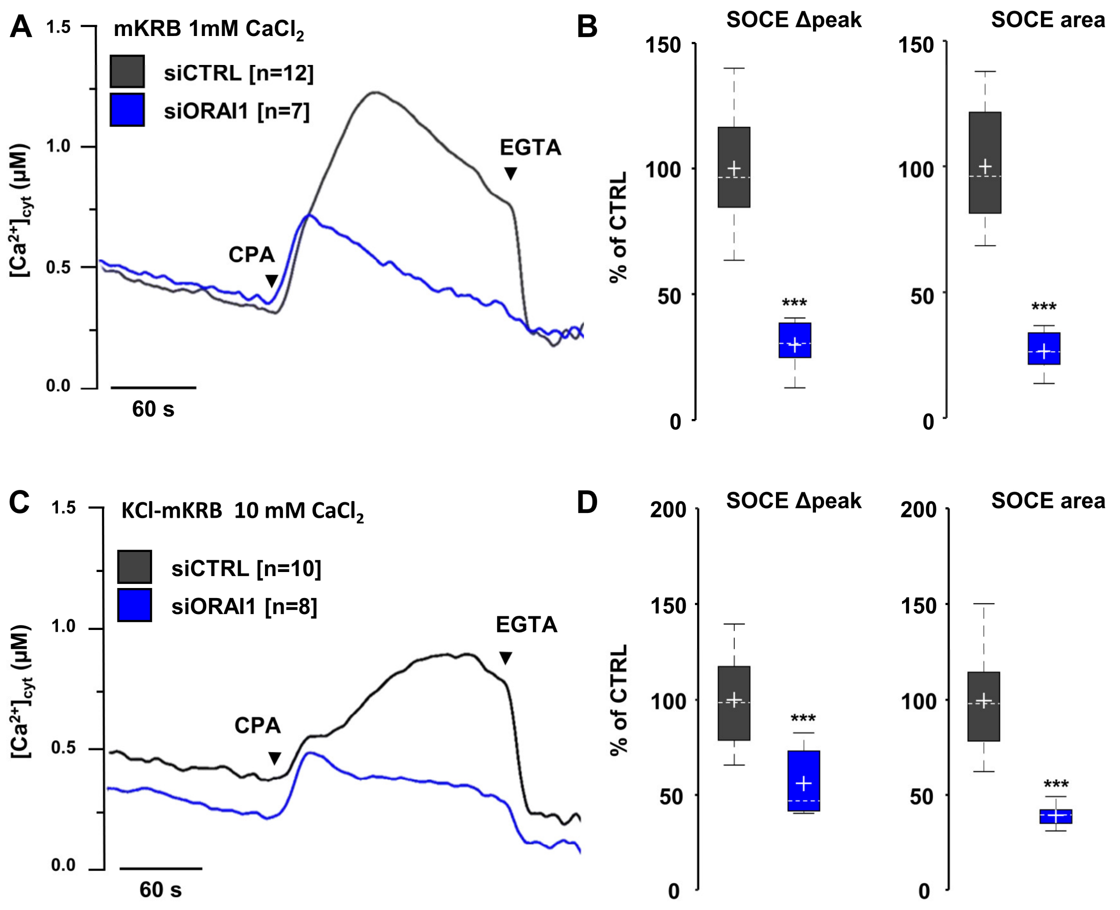

Figure S5. **ORAI1 downregulation reduces SOCE in H4-APPSwe cells.** Cells were co-transfected with siCTRL or siORAI1 plus the cDNA coding for cytAEQ. Two days later, upon cytAEQ reconstitution, cells were bathed at 37°C, for two minutes, in mKRB (**A,B**) or KCl-mKRB (**C,D**) containing 1 or 10 mM $CaCl_2$, respectively. Cells were then treated with CPA (20 µM) to activate SOCE. (**A,C**) Representative traces of siCTRL (black) and siORAI2-transfected cells (blue), respectively. (**B,D**) Boxplots of SOCE Δpeak height and AUC, measured within two minutes, upon baseline subtraction. Data are expressed as percentage of control, n = number of coverslips, Wilcoxon-Mann-Whitney test. *** $p < 0.001$.

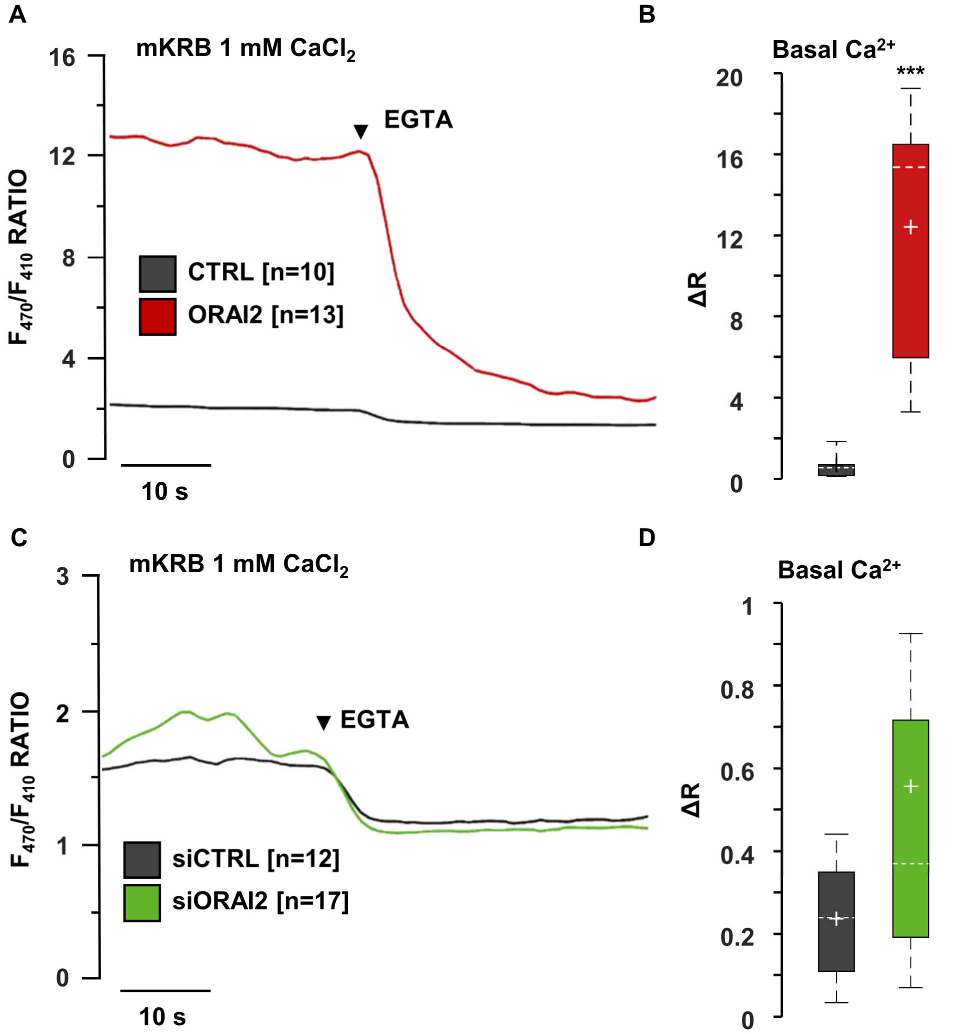

Figure S6. Effects of ORAI2 on basal Ca^{2+} levels in H4-APPswe cells. Cells were co-transfected with void-vector (CTRL) or cDNAs coding for ORAI2-myc and H2B-GCaMP6 in a 4:1 ratio (**A,B**) or with siRNAs (siCTRL or siORAI2) and the cDNAs coding for H2B-GCaMP6 (**C,D**). Cells were first bathed at 37°C in the mKRB containing $CaCl_2$ (1 mM). Differences in basal Ca^{2+} levels were evaluated by the decrease in fluorescence measured upon cell exposure to a Ca^{2+}-free, mKRB containing EGTA (0.6 mM). (**A,C**) Representative traces are shown for control (CTRL, black), ORAI2-overexpressing (red) and siORAI2-transfected (green) cells, respectively. (**B,D**) Boxplots of the difference in basal Ca^{2+} levels quantified by the ratio values in the presence and absence of extracellular Ca^{2+} ($\Delta R = R_{Ca} - R_{EGTA}$); n = number of cells from 3 independent experiments, Wilcoxon-Mann-Whitney test, *** $p < 0.001$.
